# Short period PM_2.5_ prediction based on multivariate linear regression model

**DOI:** 10.1371/journal.pone.0201011

**Published:** 2018-07-26

**Authors:** Rui Zhao, Xinxin Gu, Bing Xue, Jianqiang Zhang, Wanxia Ren

**Affiliations:** 1 Faculty of Geosciences and Environmental Engineering, Southwest Jiaotong University, Chengdu, China; 2 Institute for Advanced Sustainability Studies e. V., Potsdam, Germany; 3 Key Lab of Pollution Ecology and Environmental Engineering, Institute of Applied Ecology, Chinese Academy of Sciences, Shenyang, China; National Sun Yat-sen University, TAIWAN

## Abstract

A multivariate linear regression model was proposed to achieve short period prediction of PM_2.5_ (fine particles with an aerodynamic diameter of 2.5 μm or less). The main parameters for the proposed model included data on aerosol optical depth (AOD) obtained through remote sensing, meteorological factors from ground monitoring (wind velocity, temperature, and relative humidity), and other gaseous pollutants (SO_2_, NO_2_, CO, and O_3_). Beijing City was selected as a typical region for the case study. Data on the aforementioned variables for the city throughout 2015 were used to construct two regression models, which were discriminated by annual and seasonal data, respectively. The results indicated that the regression model based on annual data had (R^2^ = 0.766) goodness-of-fit and (R^2^ = 0.875) cross-validity. However, the regression models based on seasonal data for spring and winter were more effective, achieving 0.852 and 0.874 goodness-of-fit, respectively. Model uncertainties were also given, with the view of laying the foundation for further study.

## Introduction

With the rapid economic development of China, air pollutants are also growing rapidly in recent decades, which became one of the country’s most serious environmental issues, and attracted increasing public attention [1–3]. PM_2.5_ refers to fine particulate matter with aerodynamic diameters equal to or smaller than 2.5 microns [4], which is recognized as a major component for air pollution, which has been shown to lead to multiple adverse health outcomes [5]. A number of epidemiological studies have indicated that long-term exposure to air containing high PM_2.5_ concentrations may increase incidences of respiratory and cardiovascular diseases, and even result in death [6–8].

Generally, PM_2.5_ is monitored by ground stations, and the coverage is extended from individual points to broader planes via spatial interpolation methods such as nearest-neighbour [9] and kriging [10]. However, the monitoring results may contain uncertainties due to the limited number and uneven distribution of ground monitoring stations and sampling points for spatial interpolation [11]. To compensate for this information gap, satellite remote sensing is gradually being applied to the monitoring of air quality [12]. The aim is to establish a quantitative relationship between Aerosol Optical Depth (AOD) obtained by satellite observations and PM_2.5_ concentrations [13]. This facilitates real-time and continuous monitoring of air quality for specific regions [14].

AOD is a multiphase system formed by gases, liquid, and solid particles suspended in the atmosphere, at a scale ranging from 10^−3^ to 10^2^ microns [15]. AOD reflects the amount of transmittance through a unit section of an atmospheric column [13]. PM_2.5_ is a major component of aerosol, and is suspended in the air under a dispersed phase [16]. Studies have shown a linear correlation between PM_2.5_ and AOD [17,18].

In earlier studies, this relationship was generally established via a simplistic linear regression model between PM_2.5_ and AOD, with the understanding that PM_2.5_–AOD is stable within a certain spatiotemporal range, given as follows [19–24]:

$${\mathrm{PM}}_{2.5}=\alpha +\beta \times \mathrm{AOD}$$ (1)

where PM_2.5_ refers to its concentration near the ground (μg/m^3^), which can be measured by using the taper element oscillating microbalance (TEOM); AOD is the aerosol optical thickness (dimensionless); and *α* and *β* represent the intercept and slope, respectively.

The proposal by van Donkelaar *et al*. (2010) [17] used the ratio between PM_2.5_ and AOD for PM_2.5_ prediction. This method contained uncertainties because of insufficient PM_2.5_ data from ground monitoring [10]. After studying the relationship between aerosol and air quality in Beijing from 2005 to 2014, Chen *et al*. [25] found that there was no significant nor consistent correlation between the two, with disparities being especially large for winters and summers. This indirectly showed that improvements are needed for models that use AOD as the main variable for the prediction of PM_2.5_ concentrations.

Based on this premise, a number of studies have delineated the PM_2.5_–AOD relationship through the introduction of concomitant variables in the form of meteorological parameters, such as boundary layer height, temperature, relative humidity, and wind velocity, since the matting property of particles can drastically affect the degree of vertical mixing and increase the moisture absorption of aerosol [18, 26–28]. After modification of Eq (1), a generic model was given as follows [28]:

$${\mathrm{PM}}_{2.5}=(\alpha +\varepsilon_{1})+(\beta_{1}+\varepsilon_{2})\times \mathrm{AOD}+(\beta_{2}+\varepsilon_{3})\times \mathrm{TEMP}+(\beta_{3}+\varepsilon_{4})\times \mathrm{RH}+(\beta_{4}+\varepsilon_{5})\times SPD$$ (2)

where TEMP is temperature (°C); RH is relative humidity (%); *SPD* is wind velocity (m/s); *α* and *β* are fixed coefficients; and *ε* is a random error.

Related research has shown that organic carbides, ammonium nitrates, and sulphates are major components of PM_2.5_ [29, 30]. In addition, under certain environmental conditions, the Air Quality Index (AQI) monitoring indicators such as SO_2_, NO_2_, and CO may convert into important precursors that form PM_2.5_ [31]. Based on the data from monitoring stations in 31 Chinese cities between 2013 and 2014, Xie *et al*. [32] found a moderate correlation between PM_2.5_ and the heights at which SO_2_, NO_2_, and CO were present, but the correlation with the presence of O_3_ was weak.

Our literature review revealed that when modelling the relationship between these factors and the changes in PM_2.5_ mass concentrations, quite a few studies comprehensively considered the synergistic effects of AOD, meteorological parameters, and gaseous pollutants. In that context, this study aims to establish a quantitative model that would allow continuous monitoring of PM_2.5_ to be conducted more effectively, and that would provide insight into the spatio-temporal distribution of PM_2.5._ Herein, the multivariate linear regression method is applied, with PM_2.5_ concentration as the dependent variable, and the following as variables: AOD data, meteorological parameters (wind velocity, temperature, and relative humidity), and physical and chemical factors (SO_2_, NO_2_, CO, and O_3_).

In addition, to improve the R^2^, by means of advanced statistical models such as generalized additive regression, geographically weighted regression, and land use regression [33–35]. These studies aimed to improve model accuracy or land use information (such as altitude, population, and vegetation coverage). However, these methods could not reflect the constituent components of PM_2.5_ [36].

This paper will elaborate on the construction of the proposed model. First of all, Beijing, the capital city of China, was used as a typical case study, and all related data needed by the model for the year 2015 were collected. And then, fitting and cross-validation results of the model were obtained for the entire year and the respective seasons, before model uncertainties were discussed. The conclusion was that the constructed model could be an effective means to supplement ground monitoring for PM_2.5_ prediction. Nevertheless, there were inadequacies in the study that required further improvement.

## Methods and data source

In addition to meteorological factors, there may be different degrees of correlation between the PM_2.5_ and SO_2_, NO_2_, CO, and O_3_ [32, 37–41]. In order to verify the rationality of this conclusion, this study attempted to modify Eq (2) and construct a multivariate linear regression model. The concomitant variables of the model are the meteorological parameters and four types of pollutant indices (SO_2_, NO_2_, CO, and O_3_):

$${\mathrm{PM}}_{2.5}=(\alpha +\varepsilon_{1})+(\beta_{1}+\varepsilon_{2})\times \mathrm{AOD}+(\beta_{2}+\varepsilon_{3})\times \mathrm{TEMP}+(\beta_{3}+\varepsilon_{4})\times \mathrm{RH}+(\beta_{4}+\varepsilon_{5})\times \mathrm{SPD}+(\beta_{5}+\varepsilon_{6})\times \mathrm{CO}+(\beta_{6}+\varepsilon_{7})\times {\mathrm{NO}}_{2}+(\beta_{7}+\varepsilon_{8})\times {\mathrm{SO}}_{2}+(\beta_{8}+\varepsilon_{9})\times O_{3}$$ (3)

where PM_2.5_ is its mass concentration at ground level (μg/m^3^), AOD is derived from MODIS (dimensionless), TEMP is temperature (°C); RH is relative humidity (%), *SPD* is wind velocity (m/s); and SO_2_, NO_2_, CO, and O_3_ are the mass concentrations of the four pollutants at ground level, *β*_1_, *β*_2_, … *β*_8_ are the slopes corresponding to the respective variables; and (*α* + *ε*_1_) is the intercept.

Beijing City, which was taken as a typical case study for analysis, has seven national ground monitoring stations, including West Wanshou Nishinomiya, Temple of Heaven, Dongsi Subdistrict, Xicheng District, Agricultural Exhibition Center, The Institute of Atmospheric Physics, Olympic Centre. The data from these stations throughout the year 2015, specifically during the time period that satellite transits (i.e., AM 9:00, 10:00, 11:00, and 12:00) were obtained from the China National Environmental Monitoring Centre (http://www.cnemc.cn/), containing hourly mass concentrations of PM_2.5_ and the four major air pollutants NO_2_, CO, SO_2_, and O_3_, as well as meteorological data (including temperature, wind velocity, and relative humidity). The above data were averaged in each time node of the 4 hours respectively, to be set as the representative values for the model construction and validation. Their means, standard deviations (SD), and minimum and maximum values are shown in Table A in S1 Appendix.

In addition to ground data, this study also acquired AOD product data from the Aqua-MODIS 550 nm Collection 6. MODIS is a medium-resolution imaging spectrometer carried on the Terra and Aqua satellites of the United States’ Earth Observing System, and provides daily aerosol data worldwide [42]. The standard MODIS Level-2 (L2) product provides AOD data at 10 km spatial resolution, while the resolution of the newly-released MODIS Collection 6 (C6) product (MYD04_3K) is 3 km. In addition, the MODIS C6 product has been improved in various ways, including instrument calibration, cloud detection, the structure of the lookup table, calculation of radiation transmission, and corrections to gas absorption [43].

First, MYD04_3K data from the Aqua-MODIS 550 nm L2 Aerosol Products for the period 1 January to 31 December 2015 were downloaded through the MODIS L1 Level 1 and Atmosphere Archive and Distribution System (LAADS) (http://ladsweb.nascom.nasa.gov/). These data were verified by using the Beijing observation station’s AOD data obtained from the Aerosol Robotic Network (AERONET) (http://aeronet.gsfc.nasa.gov/). To ensure comparability between the MODIS 550 nm data and the corresponding AOD data, AERONET’s AOD data corresponding to the wavelengths 440 nm and 675 nm were interpolated to obtain the AOD value corresponding to 550 nm. The results showed a strong correlation between Aqua-MODIS 3 km AOD and AERONET AOD, with the Pearson correlation coefficient r between the two AODs being 0.92 (Fig A in S1 Appendix). The slope between the two was 1.23. This indicated that, overall, the values of MODIS AOD were higher than those of AERONET AOD. However, the deviations between the two could be considered as systematic because these were holistic and continuous throughout the entire data range [24]. As such, there would not be any impact on the prediction of PM_2.5_ concentrations.

Due to extreme weather, e.g., impact from thick clouds, strong snowfall etc. on the regional atmospheric environment, data may have deficiencies in any of the variables related to the proposed linear regression model. For example, data missing may happen to PM2.5, AOD, or the gaseous pollutants. By the reason that satellite transits within a specific time period, i.e., AM 9:00, 10:00, 11:00, and 12:00 every day in the morning, it is impossible to obtain the AOD data during the whole day. To ensure the data availability and consistency for model construction and validation, the first step is to screen out the complete dataset without deficiency for all the variables, compliance with the data capacity of AOD. As each variable has different unit, the second step is data normalization by using SPSS software to ensure them dimensionless. After data compilation and screening, 954 sets applicable to all variables were retained. To use the available data as much as possible, their ratios for the four seasons were set as the basis for stratified sampling. Two-thirds of the data were used for regression analysis at the seasonal level, while the remaining third were used for cross-validation of the model, giving 636 and 318 sets of data for annual regression analysis and model checking, respectively. Taking into account the seasonal factor, random selections were made from the 217, 239, 228, and 270 sets of data for spring, summer, autumn, and winter, respectively.

## Results and discussion

The study first gives the regression result by using the annual data of Beijing City. As the available data related to PM_2.5_ have expressed seasonal differences, they have been fitted for each season to examine their respective regression performances. The coefficients corresponding to the different regression models are shown in Table 1.

**Table 1:** The coefficients estimated in the regression models.

	Constant	AOD	Temp	RH	SPD	CO	NO_2_	SO_2_	O_3_
**Annual**	-104.046	12.21	-0.364	0.507	4.086	20.665	1.818	-0.173	0.611
**Spring**	-141.253	9.994	0.264	0.792	2.042	49.554	1.329	-0.379	1.485
**Summer**	-15.458	10.930	-0.900	-0.127	2.171	2.98	1.045	3.189	0.147
**Autumn**	-27.879	7.487	-1.258	-0.102	3.043	96.221	0.777	-3.544	0.265
**Winter**	-137.028	21.349	0.723	0.522	4.726	42.947	1.486	-1.998	0.994

## Regression results

**Fig 1:**
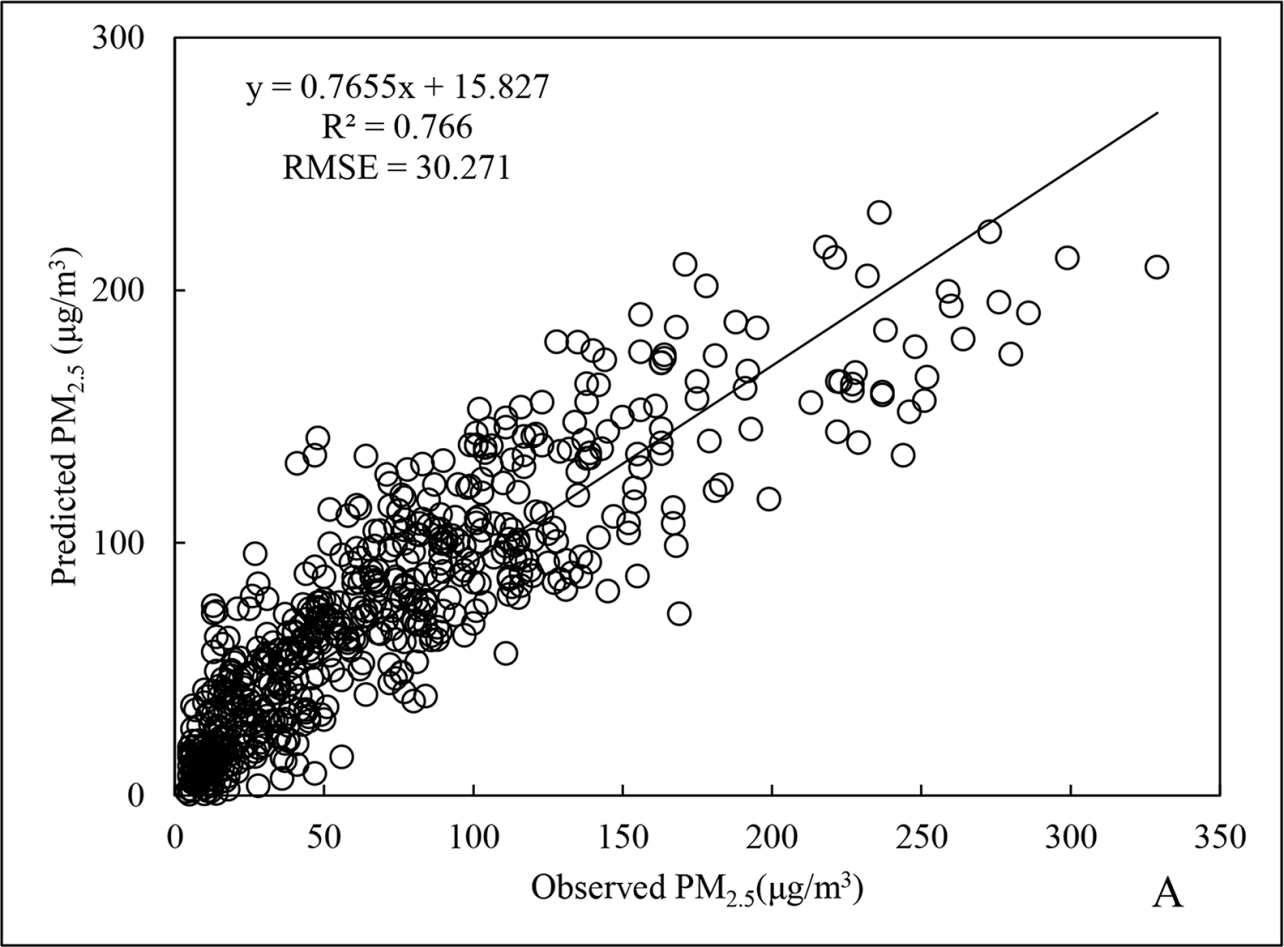
Fitting results of annual PM_2.5_ data for 2015.

**Fig 2:**
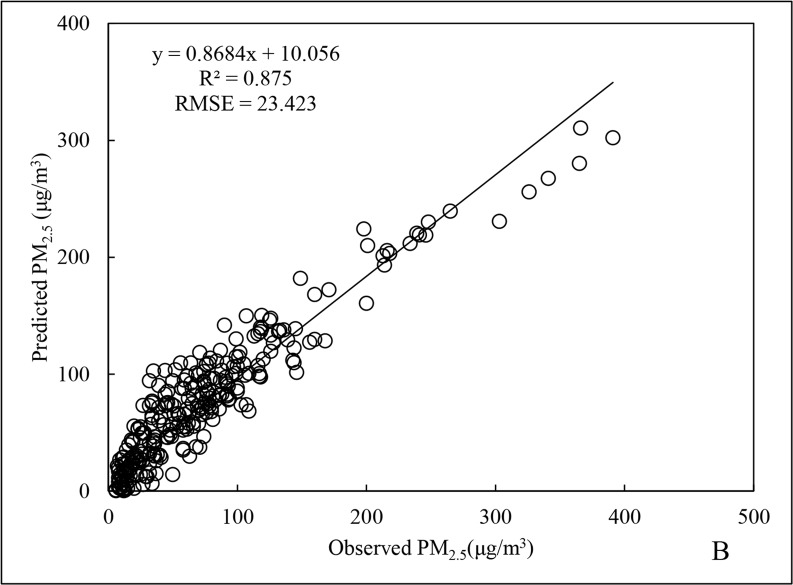
Cross-validation results of annual PM_2.5_ data for 2015.

The scatter distributions for the fitting and cross-validation of Beijing City PM_2.5_ data for 2015 are shown in Figs 1 and 2. The fitted line is generated by Excel software packaging, which is based upon the least squares method to find out the linear trend with the best fitness among the scattered points. The R^2^ and root mean square error (RMSE) for the regression model of annual data (Fig 1) were 0.766 and 30.271 μg/m^3^, respectively, and for cross-validation (Fig 2), the R^2^ and RMSE were 0.875 and 23.423 μg/m^3^, respectively, representing an increase of 14.2% and a decrease of 22.6%, respectively, compared to the regression model of annual data.

A better R^2^ results for cross-validation compared to the regression model usually might be due to the uneven distribution of sample data, which is inherent in the respective datasets for the four seasons that were used for model construction. Furthermore, the overall PM_2.5_ fluctuations between the seasons were large, such that the random selection of test data could not fully guarantee that data distribution was proportional to the actual scenarios for the four seasons.

Beijing is located in a mid-latitude region and has a temperate monsoon climate characterized by cold winters, hot summers, and distinct seasonal characteristics [44]. After the PM_2.5_ monitoring data were further grouped by the four seasons (Spring: January–March; Summer: April–June; Autumn: July–September; and Winter: October–December), the seasonality of the data was apparent. As can be seen in Table 2, the mean PM_2.5_ concentrations for spring and winter far exceeded those of summer and autumn. This observation prompted data fitting for each season, to examine the respective performance of regression. Table 3 shows that the regression results for the PM_2.5_ data in the four seasons revealed high fitness for spring and winter, both with R^2^ greater than 0.85; however, that for summer was low, with R^2^ of only 0.761. Comparison indicated that, with the exception of the data for summer, the fitting results and prediction validity for the other three seasons were all better than that of the annual data. This confirmed that the main factor affecting prediction validity was seasonal variations.

**Table 2:** Descriptive statistics and summary of regression results.

Seasonal PM_2.5_	N	Mean	SD	Min	Max
**Annual**	954	76.246	74.112	3	479
**Spring**	217	90.443	78.505	4	425
**Summer**	239	65.319	49.678	5	260
**Autumn**	228	49.639	38.364	4	199
**Winter**	270	100.688	102.127	3	479

**Table 3:** Summary of R^2^ and error measure for fitting and cross-validation result.

Seasonal Regression	Fitting results		Cross-validation results	
	R^2^	RMSE	R^2^	RMSE
**Annual**	0.766	30.271	0.875	23.423
**Spring**	0.852	29.802	0.822	21.718
**Summer**	0.761	17.977	0.618	21.082
**Autumn**	0.788	18.721	0.803	16.190
**Winter**	0.874	25.692	0.940	30.449

**Fig 3:**
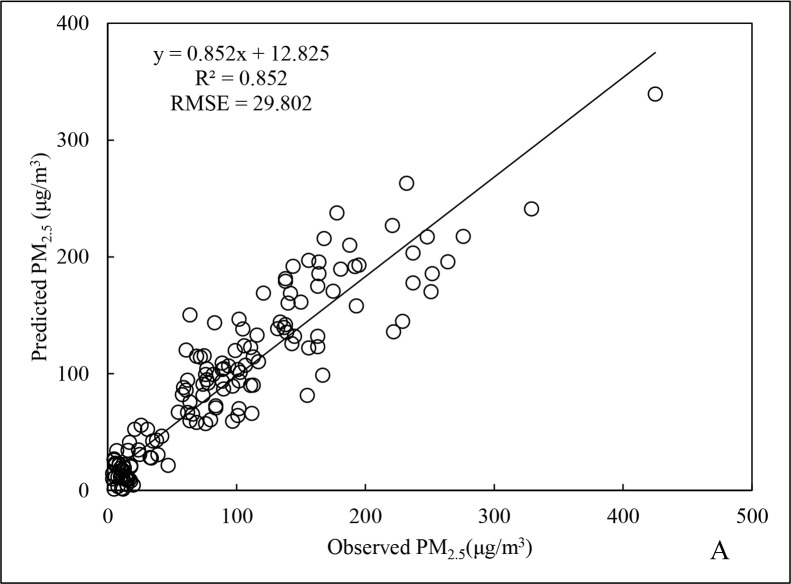
Fitting results of PM_2.5_ data for spring 2015.

**Fig 4:**
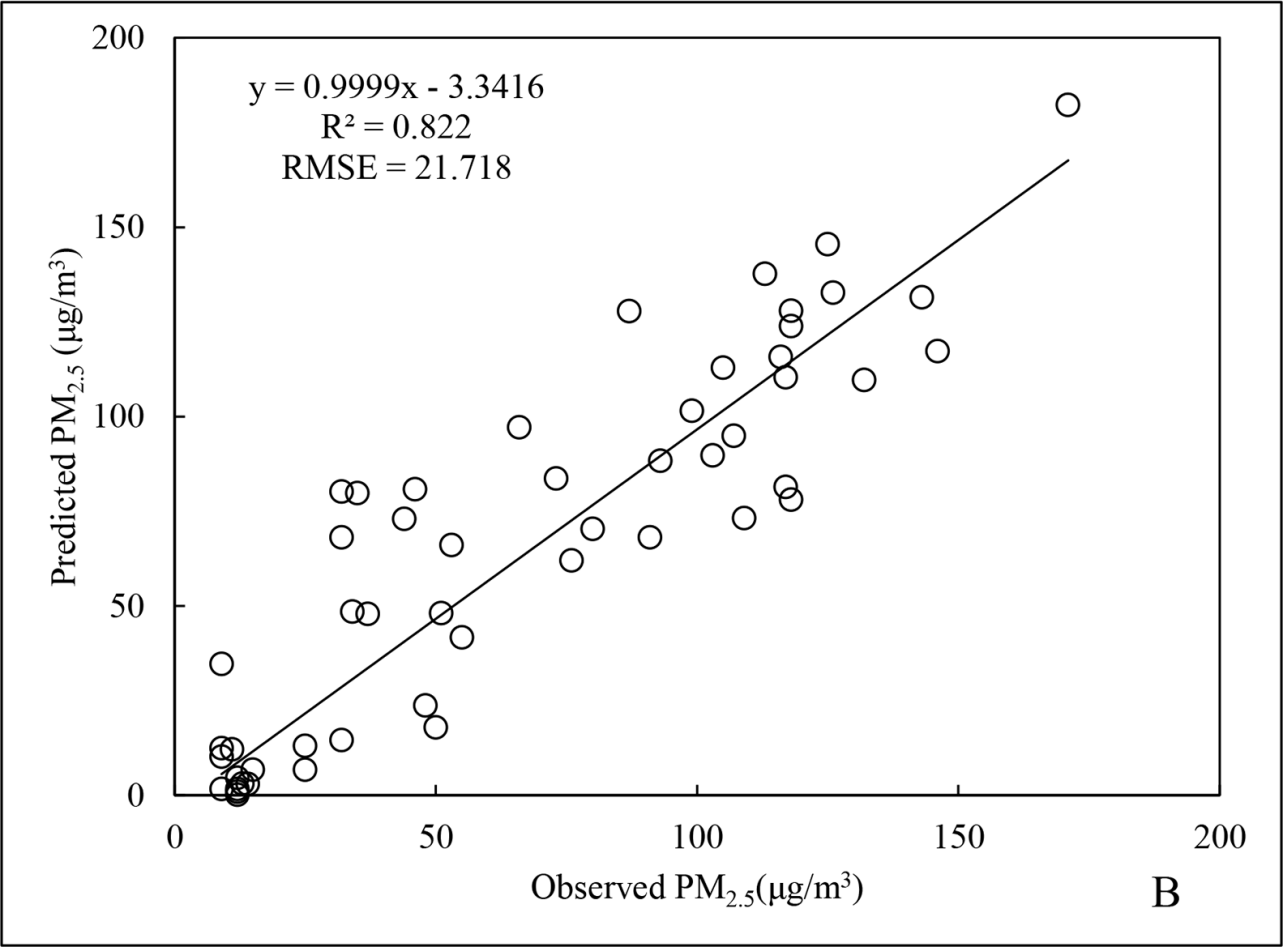
Cross-validation results of PM_2.5_ data for spring 2015.

For spring, the R^2^ for the PM_2.5_ data was 0.852 (Fig 3), representing an increase of 11.2% compared to the annual data. For RMSE, there was a decrease of 1.5% instead. The R^2^ for cross-validation was 0.822 (Fig 4), 3.5% lower than that for fitting of the spring data, indicating slight overfitting. Nevertheless, the R^2^ was still better than that for the annual data. This indirectly demonstrates that the regression model for the spring season data could accurately reflect changes in PM_2.5_ over that time period.

**Fig 5:**
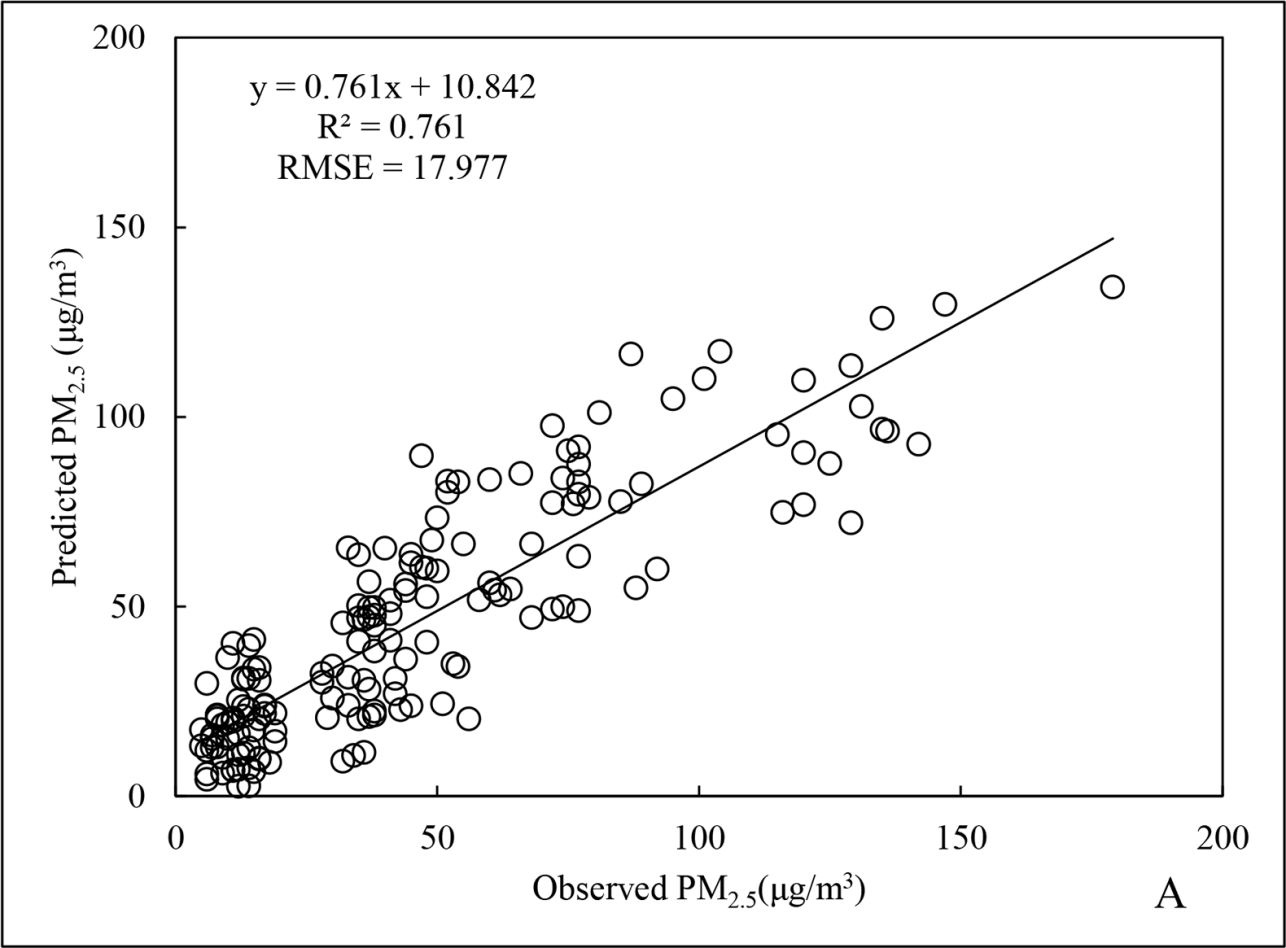
Fitting results of PM_2.5_ data for summer 2015.

**Fig 6:**
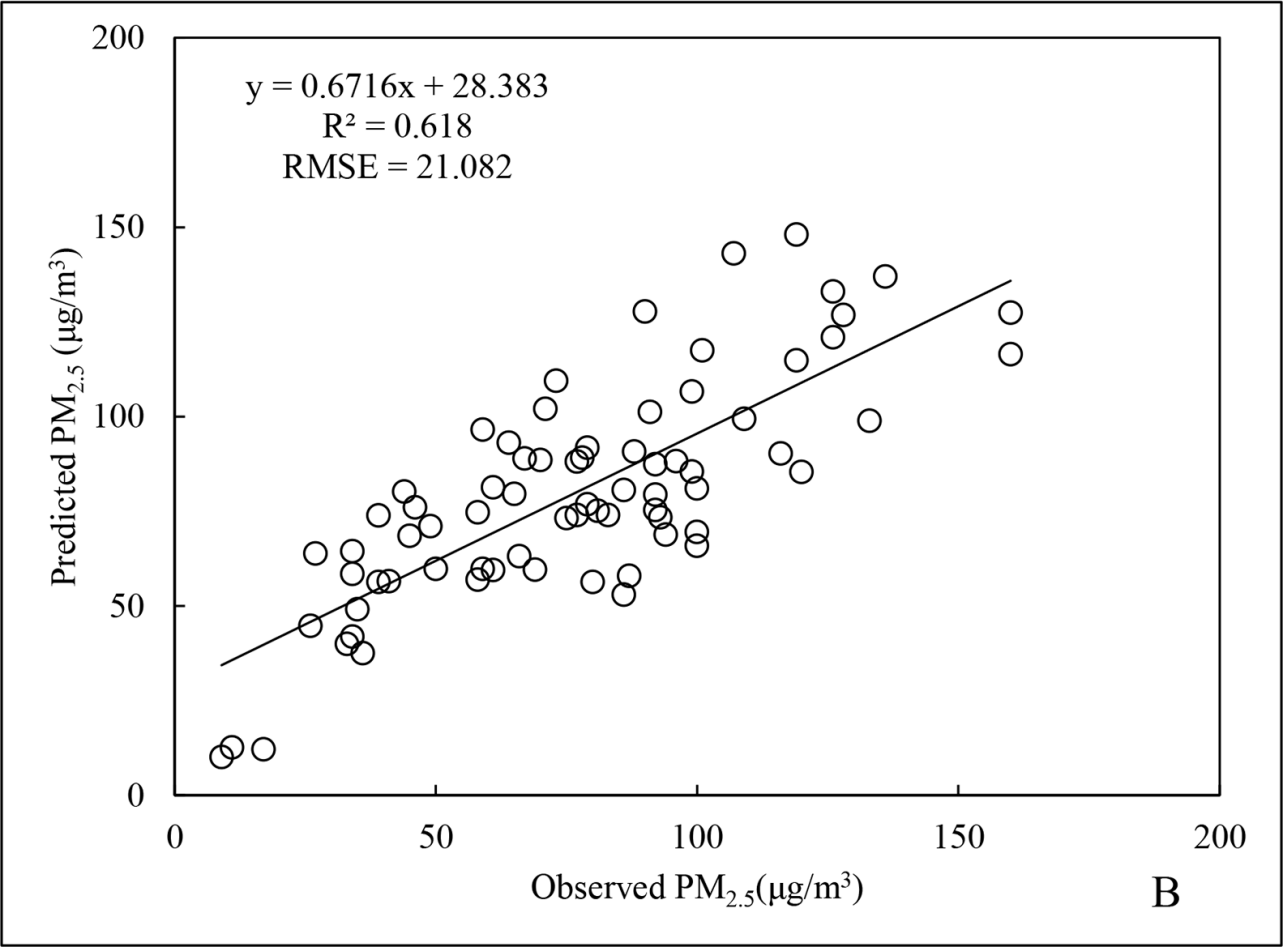
Cross-validation results of PM_2.5_ data for summer 2015.

For the summer PM_2.5_ data, R^2^ was 0.761and RMSE was 17.977 μg/m^3^ (Fig 5), being 2.4% and 40.6% lower than the results for the annual data, respectively. In addition, the R^2^ for cross-validation (Fig 6) of the summer data was 18.8% less than that for the overall data fitting, indicating slight overfitting. From the Table 1, it is apparent that the associated coefficient of carbon monoxide (CO) in summer model was much smaller than in other models. This indicated that CO had little influence on PM_2.5_ in summer, to result in an inferior performance on the summer prediction. The possible reason might be a rapid conversion of gaseous pollutants to form nitrates and sulphates, due to high summer temperatures in Beijing and longer duration of direct sunlight. Consequently, the correlation between the variable of gaseous pollutants (CO, NO_2_, SO_2_, and O_3_) and PM_2.5_ was weakened [41].

**Fig 7:**
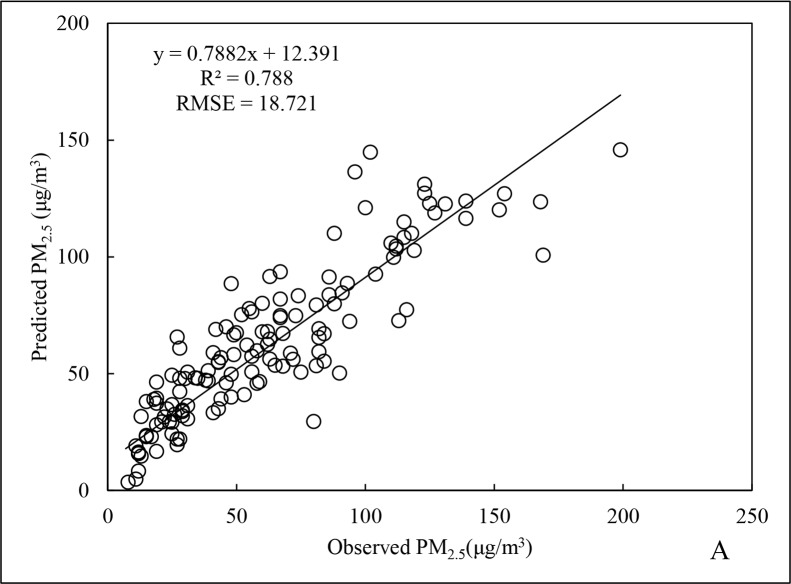
Fitting results of PM_2.5_ data for autumn 2015.

**Fig 8:**
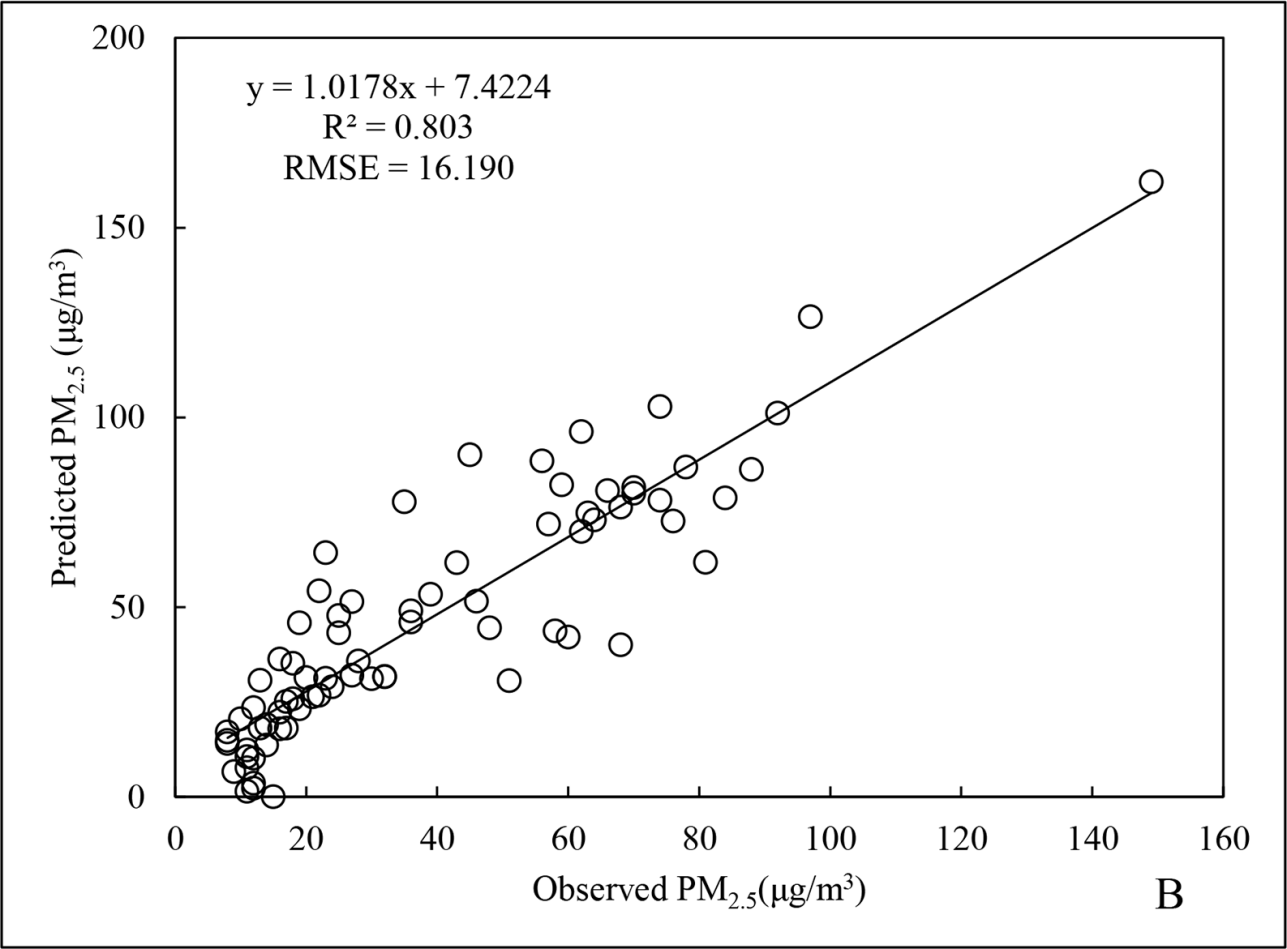
Cross-validation results of PM_2.5_ data for autumn 2015.

During the autumn, the value of R^2^ regarding PM_2.5_ data is 0.788 (Fig 7), 2.9% higher than that of annual value. In contrast, the RMSE was 18.721 μg/m^3^, being 38.2% lower than the annual data and similar to the fitting performance for the summer. The obvious reduction in RMSE might be because the mean and maximum PM_2.5_ concentrations for autumn were far lower than those for both in spring and winter. The R^2^ for cross-validation was 0.803 (Fig 8), which was slightly better than the regression result. This provides indirect evidence that the autumn data could better predict PM_2.5_ concentrations during that period.

**Fig 9:**
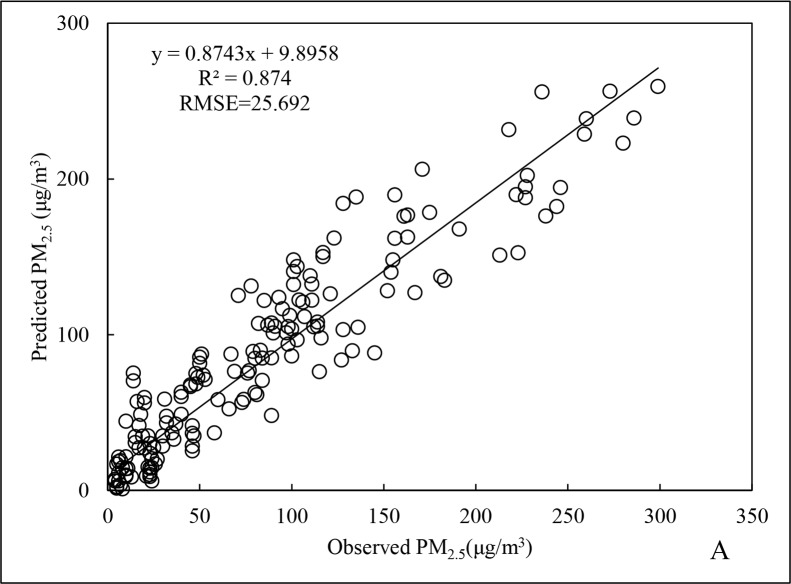
Fitting results of PM_2.5_ data for winter 2015.

**Fig 10:**
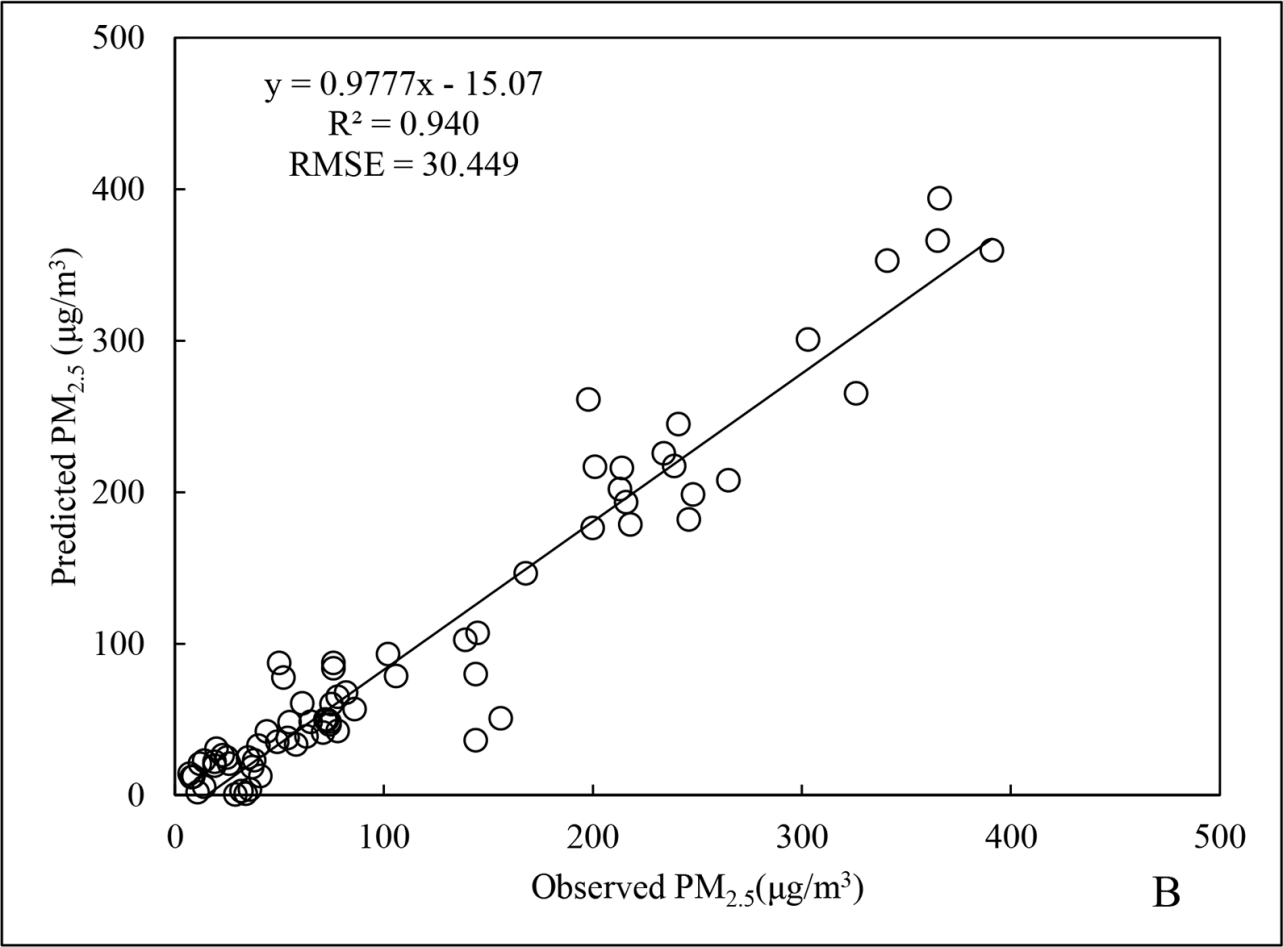
Cross-validation results of PM_2.5_ data for winter 2015.

Regarding the winter season, the R^2^ of the winter PM_2.5_ data was 0.874 (Fig 9), 14.1% higher than for the annual data. Furthermore, the RMSE was 25.692 μg/m^3^, with a decrease of 15.1%. The R^2^ for cross-validation of the winter PM_2.5_ data reached 0.940 (Fig 10), indicating that prediction validity was good. The reason would be that the mean and maximum PM_2.5_ concentrations in winter were the highest for the entire year. Meteorological conditions were stable during this period, without conducive to diffusion, resulting in good spatiotemporal stability in the data for the various parameters and variables [24].

## Discussions

Effective PM_2.5_ prediction is a complex issue that is easily affected by numerous factors, including weather and climatic conditions, and environmental seasonality. Different factors also have different degrees of impact on the regression results used for PM_2.5_ prediction. The correlation between PM_2.5_ and the other parameters and variables is shown in Table 4, and the pair-wise Pearson correlations among the parameters are given in Table B in S1 Appendix. During the study period, there was an extremely strong correlation between the mean concentration of PM_2.5_ and those of CO, NO_2_, and SO_2_, and a strong correlation between the former and AOD (highlighted in blue colour). The results reflected, to a certain extent, the rationality of the variables selected for constructing the regression model. This was especially so for regression analysis between PM_2.5_ and AOD: the model’s predictive validity improved substantially after inclusion of the four synergistic variables CO, SO_2_, NO_2_, and O_3_ to capture the contribution of gaseous pollutants to PM_2.5_ formation.

**Table 4:** Pearson correlation between PM_2.5_ and the various parameters.

	PM_2.5_	AOD	Temp	RH	SPD	CO	NO_2_	SO_2_	O_3_
**PM**_**2.5**_	1.000	0.416	-0.176	0.276	-0.134	0.784	0.772	0.500	-0.329

Separately, a comparative analysis was made between the proposed regression model and those used by other scholars. One example was the geographical weighted regression model used by Ma *et al*. [10] to predict PM_2.5_ in China, which had an R^2^ of 0.71. The prediction accuracy of the proposed model was higher (R^2^ = 0.76) than other models because the data resolution of 3 km retained much more information than studies that used data at 50 km resolution. However, this study only predicted PM_2.5_ for Beijing City, but did not take into consideration any regional differences in the atmospheric environment. To this end, it is critical to consider the spatial heterogeneity presented by PM_2.5_, as well as the impact of this heterogeneity when enhancing the model’s accuracy. The linear mixed-effect model proposed by Zheng *et al*. [45] used Beijing–Tianjin–Hebei as the study area and had an accuracy and cross-validation of R^2^ = 0.77 and 0.84, respectively. Although the fitting results were good, the model did not consider seasonal differences. Lv *et al*. [46] predicted the surface concentrations of PM_2.5_ in northern China (including Beijing, Tianjin, Hebei, and Shandong) using a Bayesian hierarchical model. The R^2^ of 0.78 was slightly better than our proposed model. A possible reason was that more AOD data were available for their use, which expanded the training set of their model, thus improving its prediction accuracy. In addition, their study took into consideration the effects of seasonal differences on PM_2.5_. This indirectly verified the rationale of our study, in which the individual seasons were used for model construction.

Although the model proposed here had higher predictive accuracy (to a certain extent) than earlier models constructed by other scholars, few uncertainties are remained. First, uncertainties in the PM_2.5_ data sources: on the one hand, this was a reflection of the uneven spatial distribution of PM_2.5_ ground monitoring stations, which were mainly concentrated in cities and urban areas, but lacking in the suburbs; and on the other hand, it revealed the systemic deviations inherent in the TEOM method of measuring PM_2.5_ concentrations [47]. Second, uncertainties in the AOD data: Although the quality of AOD products at 3 km resolution was relatively high, these were more prone to generating random noise, which affected prediction accuracies [43]. Third, uncertainties in the proposed model itself: This study only assumed a possible linear relationship between PM_2.5_ and the numerous factors but did not consider the formation mechanism of PM_2.5_, which would have an impact on the effectiveness of the model. And the fourth is the uncertainty caused by spatiotemporal heterogeneities. Depending on the region and time period, significant differences exist in PM_2.5_. The good predictions achieved by the model proposed here were limited to a region and over short duration. Further examination would be needed to determine whether the model could be applied to PM_2.5_ predictions at larger geographical scales and temporal dimensions.

## Conclusions

A multivariate linear regression equation was developed between PM_2.5_ and AOD, meteorological parameters, and various gaseous pollutants. The aim was to overcome the inadequacies in spatiotemporal observations by ground monitoring stations. The results showed that the regression model using annual data for Beijing City in 2015 could explain 76.6% of the city’s PM_2.5_ concentrations. Apparent seasonal differences in PM_2.5_ concentrations were found, with R^2^ values of 0.852, 0.761, 0.788, and 0.874 for models utilizing the data for spring, summer, autumn, and winter seasons, respectively. The results of the regression models that used spring and winter data were superior to those that used summer and autumn data. Further studies will investigate the effectiveness of the proposed model. These will include the model’s sensitivity to changes in time and region, and the effects of different resolutions of satellite-acquired AODs on prediction accuracy. The constituent components and formation of PM_2.5_ will also be analysed, so that the model can be further improved to validate its prediction.

## Supporting information

S1 Appendix (DOCX)
