# Supplementary material for: Short period PM2.5 prediction based on multivariate linear regression model
Source: PLoS One. 2018 Jul 26;13(7):e0201011. doi: 10.1371/journal.pone.0201011 (PMC6062037; doi:10.1371/journal.pone.0201011)
Supplement: S1 Appendix — (DOCX) [file pone.0201011.s001.docx]

**Supporting Information Table A. Statistics of the model parameters for 2015.**

| **Parameters** | | **Mean** | **SD** | **Min** | **Max** |
| --- | --- | --- | --- | --- | --- |
| PM_2.5_ | (μg/m^3^) | 76.246 | 74.112 | 3.000 | 479.000 |
| MODIS AOD |  | 1.101 | 0.963 | 0.065 | 5.648 |
| Temperature | (°C) | 16.843 | 11.376 | -7.000 | 40.000 |
| Relative humidity | (%) | 44.834 | 23.884 | 6.000 | 100.000 |
| Wind velocity | (m/s) | 3.426 | 2.356 | 1.000 | 14.000 |
| CO | (μg/m^3^) | 1.312 | 1.224 | 0.145 | 8.858 |
| NO_2_ | (μg/m^3^) | 49.090 | 24.825 | 4.000 | 148.000 |
| SO_2_ | (μg/m^3^) | 10.790 | 13.224 | 2.000 | 99.000 |
| O_3_ | (μg/m^3^) | 31.340 | 25.326 | 2.000 | 151.000 |

**Supporting Information Table B.** **Pair-wise pearson correlation between the parameters**

|  | | **PM_2.5_** | **AOD** | **Temp** | **RH** | **SPD** | **CO** | **NO_2_** | **SO_2_** | **O_3_** |
| --- | --- | --- | --- | --- | --- | --- | --- | --- | --- | --- |
| **PM_2.5_** | 1 | 0.416 | -0.176 | 0.276 | -0.134 | 0.784 | 0.772 | 0.500 | -0.329 |  |
| **AOD** |  | 1 | 0.216 | 0.480 | -0.286 | 0.148 | 0.245 | 0.050 | 0.129 |  |
| **Temp** |  |  | 1 | -0.028 | -0.033 | -0.358 | -0.152 | -0.454 | 0.384 |  |
| **RH** |  |  |  | 1 | -0.421 | 0.180 | 0.082 | -0.160 | -0.134 |  |
| **SPD** |  |  |  |  | 1 | -0.156 | -0.215 | -0.078 | 0.140 |  |
| **CO** |  |  |  |  |  | 1 | 0.714 | 0.610 | -0.477 |  |
| **NO_2_** |  |  |  |  |  |  | 1 | 0.582 | -0.614 |  |
| **SO_2_** |  |  |  |  |  |  |  | 1 | -0.316 |  |
| **O_3_** |  |  |  |  |  |  |  |  | 1 |  |


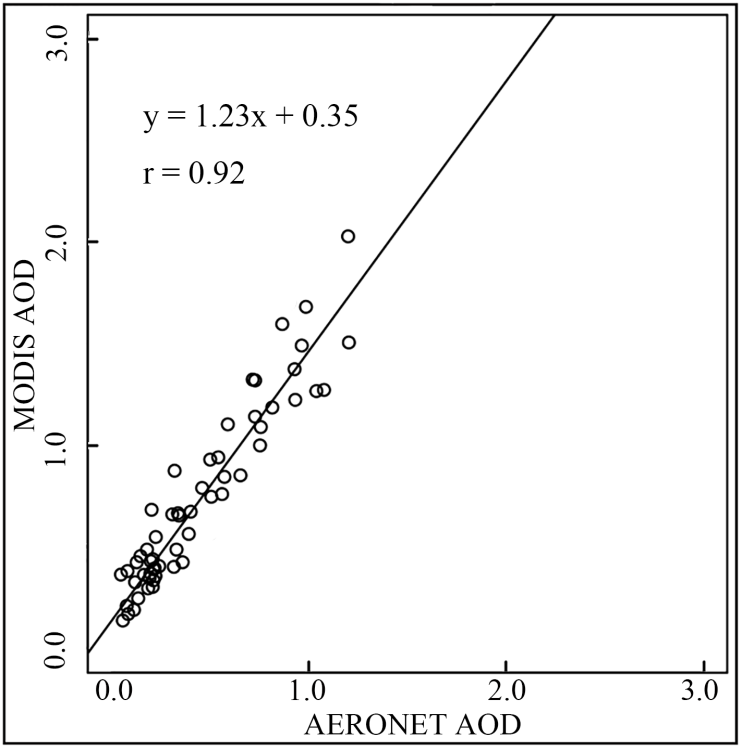


**Supporting Information Fig A. Correlation between MODIS AOD and AERONET AOD**
